# Emerging aspects of oesophageal and gastro-oesophageal junction cancer histopathology – an update for the surgical oncologist

**DOI:** 10.1186/1477-7819-4-82

**Published:** 2006-11-21

**Authors:** Ewen A Griffiths, Susan A Pritchard, Nicholas P Mapstone, Ian M Welch

**Affiliations:** 1 Department of General Surgery, The University Hospitals of Morecambe Bay NHS Trust, Royal Lancaster Infirmary, Ashton Road, Lancaster, LA1 4RP, UK; 2 Department of Histopathology, South Manchester University Hospitals NHS Trust, Wythenshawe Hospital, South Moor Road, Wythenshawe, Manchester, M23 9LT, UK; 3 Department of Pathology, The University Hospitals of Morecambe Bay NHS Trust, Royal Lancaster Infirmary, Ashton Road, Lancaster, LA1 4RP, UK; 4 Department of Gastrointestinal Surgery, South Manchester University Hospitals NHS Trust, Wythenshawe Hospital, South Moor Road, Wythenshawe, Manchester, M23 9LT, UK

## Abstract

Adenocarcinoma of the oesophagus and gastro-oesophageal junction are rapidly increasing in incidence and have a well described sequence of carcinogenesis: the Barrett's metaplasia-dysplasia-adenocarcinoma sequence. During recent years there have been changes in the knowledge surrounding disease progression, cancer management and histopathology specimen reporting. Tumours around the gastro-oesophageal junction (GOJ) pose several specific challenges. Numerous difficulties arise when the existing TNM staging systems for gastric and oesophageal cancers are applied to GOJ tumours. The issues facing the current TNM staging and GOJ tumour classification systems are reviewed in this article. Recent evidence regarding the importance of several histopathologically derived prognostic factors, such as circumferential resection margin status and lymph node metastases, have implications for specimen reporting. With the rising use of multimodal treatments for oesophageal cancer it is important that the response of the tumour to this therapy is carefully documented pathologically. In addition, several controversial and novel areas such as endoscopic mucosal resection, lymph node micrometastases and the sentinel node concept are being studied. We aim to review these aspects, with special relevance to oesophageal and gastro-oesophageal cancer specimen reporting, to update the surgical oncologist with an interest in upper gastrointestinal cancer.

## Background

In the Western world, distal oesophageal and gastro-oesophageal adenocarcinoma is increasing in incidence faster than any other type of gastrointestinal cancer [1]. During recent years there has been an increase in the understanding of these tumour types and this has implications for the histopathologist and surgical oncologist. The prognosis for patients with established cancer remains poor. However, with the increasing use of surveillance to monitor the progression of Barrett's oesophagus there is the potential for diagnosis and treatment at an earlier stage. The well defined carcinogenesis sequence of metaplasia-dysplasia-adenocarcinoma lends itself well to surveillance endoscopy.

There are many studies indicating that hospitals which manage large numbers of patients with upper gastrointestinal cancer have better outcomes [2-4], although not all evidence supports this view [5]. Upper gastrointestinal cancer services in the UK are being streamlined and reorganised with the development of hospital specialist multidisciplinary teams and regional cancer networks [6,7]. It is recommended that surgical resection for oesophagogastric cancer is performed in cancer centres serving a population of at least 1 million and containing all necessary multidisciplinary services. Although these studies favouring centralisation have largely assessed factors such as resection rates, postoperative morbidity and mortality and patient survival, there is also evidence that the quality of pathological reporting is improved [2]. Close links between the upper gastrointestinal surgeon, gastroenterologist, medical oncologist, histopathologist and other members of the multidisciplinary team are essential in improving outcomes in oesophageal cancer.

The work load of the specialist gastrointestinal pathologist is increasing, especially in the assessment of oesophageal resection specimens and endoscopic biopsy reporting. There are many reasons for this increase:

• The numbers of patients participating in Barrett's oesophagus surveillance programmes is increasing. A particularly controversial area is the designation of high grade dysplasia (HGD).

• Tumours around the gastro-oesophageal junction, which are rapidly increasing in incidence, pose several specific problems to the histopathologist.

• As with other tumour sites, standardisation and an emphasis on quality and completeness of pathological reporting have become mandatory. There is a need for pathologists to comply with the criteria in the minimum dataset for reporting oesophageal cancer specimens.

• In some units the pathologist has a role in the assessment of the resected specimen immediately after surgical excision.

• Newer evidence on the importance of several histopathological prognostic factors, including circumferential resection margin status and lymph node metastases, will have special implications for specimen reporting.

• With the rising rates of multimodal treatments for oesophageal cancer the pathologist will have an increasing role in documenting the response of the tumour to this therapy.

• The issues of endoscopic mucosal resection (EMR), lymph node micrometastases and the sentinel node concept have the potential to expand the scope of the histopathologist.

We aim to review these aspects, with special relevance to oesophageal and gastro-oesophageal cancer specimen reporting, to update the surgical oncologist with an interest in upper gastrointestinal cancer.

### Endoscopic biopsies

Accurate assessment of endoscopic biopsy material is crucial in the assessment of patients with Barrett's oesophagus, oesophageal epithelial dysplasia and adenocarcinoma. The British Society of Gastroenterology (BSG) has recently published guidelines for the diagnosis and management of Barrett's columnar-lined oesophagus [8]. More biopsies are being examined due to the increase in number of patients enrolled on endoscopic surveillance programmes. Accurate classification into these diagnostic categories often requires multiple biopsies, especially in high-grade dysplasia (HGD). The Seattle group recommend four quadrant biopsies for every 2 cm interval of Barrett's change identified at screening endoscopy [9]; this is increased to four quadrant biopsies every 1 cm interval in cases of follow-up of Barrett's dysplasia. A thorough review of the current pathological aspects of these pre-malignant changes is beyond the scope of this review (for recent review see [10]). However, the controversy surrounding Barrett's dysplasia, especially HGD, deserves a mention.

#### Barrett's dysplasia

Dysplasia is defined as unequivocal neoplastic transformation of the epithelium, strictly confined within the basement membrane of the gland from which it arises [11]. There is frequent disagreement between the classification of HGD and intra-mucosal carcinoma [12]. The WHO recommend the use of high grade intra-epithelial neoplasia to cover both HGD and carcinoma *in-situ *to try to increase inter-observer agreement, however in the UK dysplasia is still in use. Changes in the epithelial cells include lack of maturation towards the surface, variation in nuclear size and shape, nucleolar enlargement, increased cytoplasmic ratio, hyperchromasia and presence of abnormal mitoses. Architectural changes seen in dysplasia include stratification of nuclei with loss of the normal basal location. Barrett's dysplasia is classified into either indefinite for dysplasia, low grade dysplasia (LGD) or HGD by the degree of cellular and architectural changes [13,14]. A diagnosis of indefinite for dysplasia is made when histological features suggestive of dysplasia are seen but the presence of inflammation makes it impossible to distinguish confidently between reactive changes and true dysplasia. The BSG guidelines recommend prompt follow-up and treatment of cases of indefinite dysplasia with acid suppression followed by extensive endoscopic biopsies [8]. Further endoscopy and biopsies should then be taken after six months and if all fail to show definite evidence of dysplasia the patient can return to routine follow-up. LGD should be managed by acid suppression for eight to twelve weeks followed by repeat endoscopy and extensive re-biopsy. If the LGD persists surveillance should be six monthly. If regression occurs on two consecutive examinations surveillance intervals may be increased to two to three yearly.

As a consequence of the subtle cytological changes from LGD to HGD, previous studies have shown that there are marked intra-observer and inter-observer variations in the classification of the degree of dysplasia [15]. The clinical application of these studies is to emphasize the need of a second opinion from an experienced gastrointestinal histopathologist in difficult cases, especially when the distinction is clinically important and will change therapeutic management [16]. This may require a further endoscopy for collection of additional material using the 'Seattle' protocol to ensure sufficient tissue for accurate designation is available and to minimise the risk of missing an occult adenocarcinoma [9]. Unfortunately there are at present no reliable immunohistochemical markers available to distinguish between reactive changes and dysplasia. When there is consensus among three pathologists on the designation of LGD, then the progression to HGD or carcinoma appears to be substantial [17].

Appropriate care of patients with HGD in the setting of Barrett's oesophagus relies heavily on the accuracy of reporting the degree of dysplasia [18]. Early studies have shown that HGD was associated with up to a 73% risk of undetected adenocarcinoma on subsequent oesophagectomy specimens [19,20]. There are conflicting results on whether the risk of progression is related to the extent of HGD present. In one study, the risk was unrelated to the amount of HGD present [21]; therefore even a small area should not be discounted. However, in another study multifocal HGD had a higher risk of progression to malignancy than unifocal HGD [22]. This may be due to factors relating to genomic instability and clonal expansion [23].

Treatment options for HGD are controversial and range from intense endoscopic surveillance, endoscopic ablative therapy, EMR and oesophagectomy [24]. In those unfit for surgery endoscopic ablation or EMR should be considered. In Japan, EMR is the standard treatment for early neoplasms but these tend to be squamous cell carcinomas which have many differing characteristics to adenocarcinomas. The recent BSG guidelines recommend oesophagectomy in a specialised unit in patients considered fit for surgery.

### Standardisation of oesophageal resection specimen reporting

The histopathologist has an important role in ensuring quality and a consistent approach to pathological reporting. An accurate histopathology report is essential for providing detailed staging of the primary tumour, elucidating prognostic information and for guiding optimal clinical management decisions. Audit, research, cancer registry data and other epidemiological studies rely heavily on the accuracy of this information. Upper gastrointestinal surgeons and clinicians have demanded an increase in the quality and quantity of information from the pathologist.

The Royal College of Pathologists have published guidelines on the standards of oesophageal resection specimen reporting which include a proforma detailing the Minimum Dataset requirements [25]. A similar 'best practice' report has been published by the Association of Clinical Pathologists (ACP) [26] and the College of American Pathologists has its own guidelines [27]. Despite this guidance the quality of pathological reporting has been variable [28,29]. Missing data items from an audit of oesophageal resection specimens reported in 2004 included key data (% missing) such as tumour differentiation (14%), proximal margin involvement (17%), distal margin involvement (19%), completeness of resection (48%) and circumferential margin involvement (48%) [29]. It is hoped, as with other cancer types, that the increased use of proformas will increase quality and standardisation of specimen reporting [28]. The centralisation of oesophageal surgery services in one region resulted in a significant improvement in oesophageal resection specimen reporting [2].

### The resected specimen: problems around the gastro-oesophageal junction (GOJ)

Tumours around the gastro-oesophageal junction (GOJ) have become commoner in recent decades [1,30,31] and present particular challenges. Classification systems for GOJ tumours have been devised [32,33], but sadly they have not been widely adopted into routine clinical practice in the UK. Sub-classification is not a part of the current requirements of the Royal College of Pathologists Minimum Dataset for reporting oesophageal cancer [25]. Here a carcinoma is classified as oesophageal if more than half of the tumour is above the gastro-oesophageal junction. Histopathologists sometimes find the distinction between oesophageal and gastric tumours surrounding the gastro-oesophageal junction difficult. This is usually defined endoscopically as the upper limit of the gastric rugal folds. In large tumours the gastro-oesophageal junction may be obliterated making it impossible to comment on whether the tumour is mainly above or below it. In such cases the anatomical site (recognised by the peritoneal reflection at the junction of the oesophagus and greater curve of the stomach) of the gastro-oesophageal junction may be of help [26]. The histopathologist may look for the presence of columnar lined oesophagus above the tumour that would be suggestive of an oesophageal origin, especially if associated with dysplasia, or for gastric dysplasia suggesting gastric origin. However, the definition, location and extent of the gastric cardia and GOJ are controversial in much of the medical literature. This can make it harder to compare and contrast previously published studies including different patient populations with heterogeneous tumour types.

**Figure 1:**
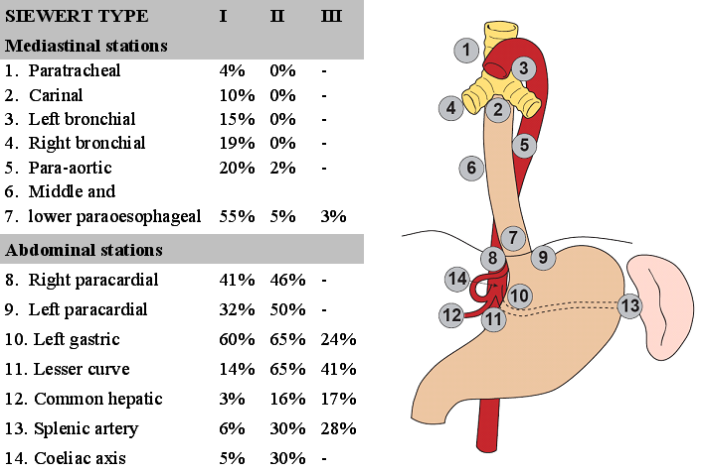
The lymph node stations surrounding the oesophagus and upper stomach are shown. Type I, II and III gastro-oesophageal tumours vary in their predilection for involvement of different lymph node stations in the mediastinum and abdomen. Please note that the information about metastatic spread to the mediastinal lymph nodes in Type III tumours is limited as surgical resection does not normally include these nodes. Information on percentage of lymph node metastases taken from Dresner et al [110] and Ichikura et al [121].

The UICC/AJCC classification systems rely on the anatomical location of the epicentre or predominant mass of the tumour to decide whether the tumour is oesophageal or gastric in origin [34]. With the increasing proportion of these cancers, especially types which straddle the GOJ with equal proportions on each side, it has become apparent that this system is inadequate. The Siewert classification system was approved following a consensus conference of the International Society for Diseases of Esophagus (ISDE) meeting in 1995 [35] and is the most widely adopted classification system. GOJ tumours are anatomically classified into three sub-types depending on distance from the cardia, which is defined as the proximal end of the typical longitudinal gastric mucosa folds [33]. According to the authors, there are key differences in epidemiological, clinical and pathological characteristics between these tumour subtypes and the classification system can also aid planning of surgical treatment (Table 1). These tumour sub-types also differ in their predilection for lymph node metastases to the mediastinal and abdominal lymph node stations (Figure 1). Siewert's classification system is recommended for use by the British Society of Gastroenterology in the published *Guidelines for the management of oesophageal and gastric cancer *as 'it is uniform, allows data comparison from different centres, and is important for the stratification of patients in prospective studies' [16]. Criticisms of the Siewert system are 1) that it unnecessarily complicates the assessment of these tumours; 2) it is difficult to assign some tumours because of their size and overlapping nature and 3) that some authors prefer to classify distal oesophageal and gastroesophageal junction tumours as one clinical entity as they have found similar distributions of lymph node metastases and survival [36-38].

**Table 1:** Tumour around the gastro-oesophageal junction: classification system and principal differences. (Information taken from [33, 42, 110])

**GOJ subtypes**	**Type I (Adenocarcinoma of distal oesophagus)**	**Type II (True cardia carcinoma)**	**Type III (Sub-cardial carcinoma)**
**Endoscopic criteria**	Tumour mass arises 1 to 5 cm above the endoscopic cardia	Tumour mass arises 1 cm above to 2 cm below the endoscopic cardia	Tumour mass arises 2 to 5 cm below the area of the endoscopic cardia
**Differing Characteristics**	• Male predominance • Arise in association with Barrett's oesophagus (80%) • More likely to have hiatus hernia or history of GORD	• More similarities to Type III tumours than Type I • Barrett's mucosa identified in 10%	• Barrett's mucosa identified in only 2% • 60% have a diffuse growth pattern and 70% undifferentiated
**Lymph node metastases**	To mediastinal and abdominal lymph node stations	Mainly to abdominal lymph node stations	Mainly to abdominal lymph node stations
**Precursor lesions**	Barrett's oesophagus	Possible short segment Barrett's oesophagus or IM at the gastric cardia	Helicobacter pylori and IM of the subcardia region
**Optimal surgical treatment**	Transthoracic or transhiatal oesophagectomy	Controversial; may include either extended total gastrectomy or transthoracic or transhiatal oesophagogastrectomy	Extended total gastrectomy

IM = intestinal metaplasia, GORD = gastro-esophageal reflux disease

### Problems with the current TNM staging systems

The existing TNM staging system also has some deficits [39], which include the following:

• It is based primarily on data from patients with squamous cell carcinoma of the middle and upper oesophagus

• There is confusion regarding whether the oesophageal or gastric TNM systems should be used for GOJ tumours

• Lymph node involvement beyond the regional lymph nodes is considered metastatic disease (M1)

• The number of positive lymph nodes has been shown to be a strong prognostic factor by many authors, however, this is not apart of the current TNM system

Currently there is confusion about whether the gastric or oesophageal staging systems should be used for the histological reporting of gastro-oesophageal junction tumours. Due to this confusion a tumour at the GOJ could be classified as oesophageal by the surgeon but gastric by the histopathologist or vice versa, especially when cases are not discussed at a preoperative multidisciplinary meeting. Usually, Type I adenocarcinomas are staged as oesophageal cancer and Type III as gastric cancer. Type II adenocarcinomas are staged as oesophageal cancer by some authors and as gastric by others. This is not ideal as there are significant differences between the gastric and oesophageal TNM staging systems in all three TNM categories (Table 2). There are major differences in the pT stages between the two systems, but the most significant differences are in the pN staging category. In oesophageal cancer nodal involvement is merely classified as nodal positive (pN1) and nodal negative (pN0), irrespective of the number of lymph nodes involved. In comparison, for gastric cancer TNM staging, the pN category is sub-classified according to the number of involved nodes: pN1 (1–6 positive nodes), pN2 (7–15 positive nodes) and pN3 (>15 positive nodes). Metastatic lymph nodes to the coeliac axis are classified as systemic spread (pM1a) in the oesophageal system, whilst they are classified as regional in the gastric staging system. Therefore, the classification of Siewert type II tumours with positive celiac lymph nodes is controversial. Similarly, metastases to the supra-diaphragmatic nodes or to the nodes of the lower mediastinum are considered 'non-regional' in gastric cancer, and are classified as distant metastases (pM1) in type II and type III junctional tumours. Consequently there have been calls for tumours around the GOJ to have a separate TNM staging system [40]; however this remains to be designed or approved by the UICC/AJCC.

**Table 2:** Comparison between the oesophageal and gastric TNM staging [34]

		**Oesophageal staging**		**Gastric staging**
**T Stage**	**T0**	No evidence of primary tumour	**T0**	No evidence of primary tumour
	**Tis**	Carcinoma in situ	**Tis**	Carcinoma in situ
	**T1**	Tumour invades lamina propria or submucosa	**T1**	Tumour invades lamina propria or submucosa
	**T2**	Tumour invades muscularis propria	**T2a**	Tumour invades muscularis propria
	**T3**	Tumour invades adventitia	**T2b**	Tumour invades subserosa
	**T4**	Tumour invades adjacent structures	**T3**	Tumour penetrates serosa (visceral peritoneum) without invasion of adjacent structures
			**T4**	Tumour invades adjacent structures
**N Stage**	**NX**	Regional lymph nodes cannot be assessed	**NX**	Regional lymph nodes cannot be assessed
	**N0**	No regional lymph node metastases	**N0**	No regional lymph node metastases
	**N1**	Regional lymph node metastases	**N1**	Metastases in 1 to 6 regional lymph nodes
			**N2**	Metastases in 7 to 15 regional lymph nodes
			**N3**	Metastases in more than 15 regional lymph nodes
**M Stage**	**MX**	Distant metastases cannot be assessed	**MX**	Distant metastases cannot be assessed
	**M0**	No distant metastases	**M0**	No distant metastases
	**M1a**	Metastases to coeliac or cervical lymph nodes	**M1**	Distant metastases
	**M1b**	Other distant metastases		

### Histopathological prognostic markers in oesophageal and GOJ cancer

The most important predictors of prognosis appear to be the overall TNM stage [34], completeness of resection (R classification) [41,42] and the presence of lymph node metastases [43]. Other important histopathological factors are summarised in Table 3. Although newer molecular based markers are being assessed to see if they can be used to predict prognosis, none are in routine clinical use and they are beyond the scope of this article (for recent review see [44]).

**Table 3:** Histopathological prognostic factors after surgical resection of oesophageal cancer

**Factor**	**Reference**
Residual (R) tumour classification *	[41, 42]
Proximal and distal margin involvement	[46, 47]
Circumferential resection margin involvement	[50, 51, 56]
Tumour invasion (T stage)	[42, 68, 111, 112]
Lymph node metastases	[42, 43, 68]
Vascular invasion	[53, 113, 114]
Lymphatic vessel invasion (LVI)	[61-63]
Perineural invasion	[115]
Tumour length	[43, 116]
Tumour differentiation	[117, 118]
Histological subtype	[65]

* R0 complete microscopic and macroscopic resection; R1 residual microscopic disease; R2 residual macroscopic disease

#### Residual disease classification and resection margin involvement

The residual tumour classification (Table 4) is one of the strongest prognostic factors after surgical resection [41,42]. It is classified into: R0, complete microscopic and macroscopic resection; R1, residual microscopic disease and R2, residual macroscopic disease (Table 4) [45]. Both the pathologist and the surgeon have roles in defining the R status during and after surgery.

**Table 4:** Residual (R) tumour classification system [45]

**R Classification**	**Meaning**
**R0**	Complete resection of microscopic and macroscopic disease
**R1**	Incomplete resection; residual microscopic disease
**R2**	Incomplete resection; residual macroscopic disease

The circumferential resection margin (CRM) is defined as the surgically cut surface of the connective tissues that encase the oesophagus. Presence of tumour within 1 mm of this resection margin is classified as evidence of involvement. Although it has long been established that involvement of the proximal or distal resection margin is a poor prognostic factor [46,47], the relevance of the CRM status has been unclear and few studies have addressed this issue. However, the increased awareness of the CRM status in rectal cancer has inspired investigation in oesophageal cancer. In rectal cancer surgery, the pathological reporting of CRM is important as its status predicts risk of local disease recurrence and reduced survival [48,49]. It is routinely reported in all rectal resection specimens and involvement is often an indication for post-operative chemotherapy and/or radiotherapy.

#### Circumferential resection margin (CRM) involvement and oesophageal cancer reporting (Table 5)

**Table 5:** Studies assessing the prognostic impact of CRM status in oesophageal cancer

**Author, Date**	**No.**	**Country**	**Type study**	**Tumour**	**% CRM involvement**	**Significance on univariate survival**	**Significant on multivariate survival**
Sagar, 1993 [50]	50	UK	R	Adeno, SCC	40%	Yes (p < 0.05)	Not tested
Saha, 2001 [56]	59	UK	R	Adeno	Unknown	Yes (p < 0.01)	Yes (p < 0.05)
Dexter, 2001 [51]*	135	UK	P	Adeno, SCC	47%	Yes (p < 0.015)	Yes (p = 0.013)
Zafirellis, 2002 [53]*	156	UK	P	Adeno, SCC	42%	Yes (p < 0.0001)	Not significant
Khan, 2003 [54]	329	UK	R	Adeno, SCC	20%	No (p = 0.57)	Not applicable
Roh, 2004 [119]	59	Korea	R	SCC	44%	Yes (p = 0.003)	Not tested
Griffiths, 2006 [52]	249	UK	R	Adeno, SCC	32%	Yes (p = 0.0001)	Yes (p = 0.007)

R = retrospective, P = prospective, Adeno = adenocarcinoma, SCC = squamous cell carcinoma, *These two studies from the same research group include some of the same patients.

An initial study by Sagar *et al *[50] in 1993 assessed 50 patients undergoing oesophagectomy and found that cancer involvement of the CRM was associated with increased risk of local disease recurrence and significantly reduced 2-year survival. More recently, Dexter *et al*., studied 135 patients who underwent oesophagogastrectomy [51], they included only the patients who had underwent a potentially curative procedure, excluding patients with other margin involvement, T4 tumours, M1a or M1b disease and palliative resections. The rate of CRM involvement in their study was 47%. Survival was significantly reduced in patients with CRM involvement who would have been otherwise considered to have had a potentially curative resection. CRM involvement was also an independent predictor of survival on multivariate analysis. They also observed that when they stratified patients into low and high nodal metastatic burden (< or > 25% involved lymph nodes), the effect of CRM status on survival was more significant in patients with a low ratio of involved metastatic lymph nodes. A recent report suggested that even in patients with T3 tumours and a low percentage of lymph node metastases (<25%) there is an improved prognosis if the CRM were negative [52].

In a follow-up study with larger patient numbers carried out by the same group [53], CRM was still a prognostic factor on univariate analysis but lost its significance as an independent prognostic variable. However in this study, the R classification (which included CRM status) was an independent prognostic factor, together with nodal status and vascular invasion.

Not all studies have shown a positive CRM to predict a poor prognosis in oesophageal cancer. Khan *et al*., [54] observed 329 patients undergoing resection and found no difference in survival between patients with or without CRM involvement. Although the reasons for the difference are not entirely clear, the surgical technique in the Khan study favoured extensive mediastinal dissection. For example, in this study only T3 tumours (tumour invading the adventitia) had evidence of CRM involvement. In the Dexter study there were cases of T2 tumours (tumours invading muscularis propria) involving the CRM [51], suggesting that less radical surgery was performed. In support of this, the study by Khan *et al*., had the lowest rate of CRM involvement (20%). Therefore the prognostic impact of CRM status may be related to the completeness of the mediastinal dissection. A similar situation is present in rectal cancer surgery, where the prognostic effect of CRM involvement is lessened following more radical surgical resection [55].

Although Khan *et al*., questioned whether CRM status should be an essential part of oesophageal resection specimen reporting [54], the majority of studies support the notion that CRM involvement is a significant prognostic factor [50-53,56]. Moreover, three of these studies have shown it to be a significant independent predictor of survival on multivariate analysis [51,52,56]. It would seem sensible to continue to record the involvement of the CRM in oesophageal resection specimen reports.

#### CRM status as a marker of quality of surgery

It is important that the anatomy of the oesophagus is understood, especially as regards to the surrounding structures in the thorax. The CRM includes the whole circumference at and just above the gastro-oesophageal junction, but more proximally it is concentrated anteriorly and posteriorly with pleura on the lateral aspects. The pleural surfaces are not a true CRM and the significance of tumour involvement at this site is uncertain as there have been no large studies examining this [57]. It can be difficult to identify the pleura on the resected specimen but this difficultly is removed if the surgeon marks the pleural surface or if the fresh resected specimen is seen and discussed together by both surgeon and pathologist.

**Figure 2:**
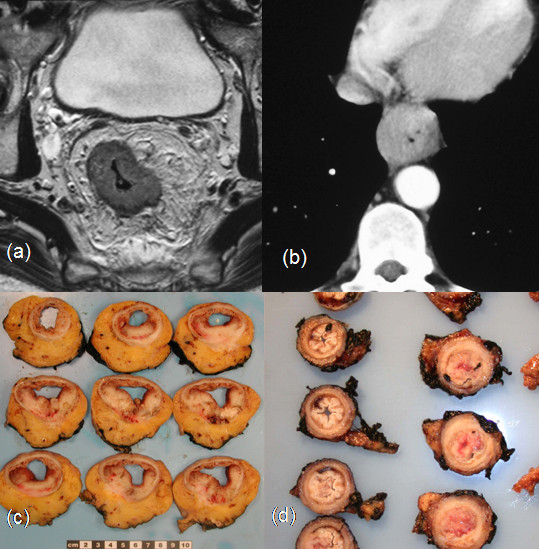
(a) MRI of the pelvis showing the ample surrounding mesorectal tissue and fascia, in comparison to (b) which shows a CT image of the mediastinum showing how little tissue separates the oesophagus from important unresectable structures such as the aorta and heart. (c) Transverse cut sections of anterior resection specimen for rectal cancer (note how much more surrounding tissue there is compared with the oesophageal specimen) (d) Transverse cut sections of oesophageal resection specimen; this method of sectioning allows direct comparison with the pre-operative staging.

In rectal surgery, a large intact capsule of mesorectum surrounding the resected cancer specimen has been suggested as a marker of good quality surgery [58,59]. However, there are important anatomical differences between the pelvis and the mediastinum (Figure 2). The oesophagus lacks a serosal layer, so that tumours originating in the oesophagus can easily spread into several important structures (pericardium, heart, great vessels, trachea and lung). The majority of these adjacent structures cannot be sacrificed and resected *en-bloc*. In addition, there is no specific fascial boundary equivalent to the mesorectal or Denonvillier's fascia present in the pelvis. CRM involvement in the oesophageal cancer specimen may therefore be much more a consequence of advanced tumour stage rather than the skill of the surgeon in carrying out a complete resection. Nevertheless, involvement of the CRM may be an indicator of the quality of pre-operative staging. At present most pathologists open oesophagogastric resections longitudinally, with or without pinning out of the fresh specimen, for fixation [26]. This does not allow good comparison with radiological imaging. In rectal tumours transverse slices through the tumour are advocated to allow comparison with MRI. If the quality of oesophagogastric cancer preoperative staging is to be studied transverse slices through the tumour are recommended. The fact that some surgeons remove lymph nodes from the main specimen and submit them separately prevents assessment of the CRM; this should be discouraged. All of the information acquired by separate dissection of the lymph nodes should be available from thorough, although time consuming, macroscopic examination of the fixed specimen. Some feel that separate dissection of the lymph nodes yields a higher lymph node count compared to lymph node harvesting by the histopathologist. However serial slicing with narrow slice width and careful examination of all attached fat, both by palpation and visually, should result in a high lymph node yield. It may be useful to take a macroscopic photograph of the sliced specimen to compare with preoperative imaging.

#### Serosal invasion

Serosal invasion is an important prognostic marker in many gastrointestinal malignancies [57]. Gastro-oesophageal junctional tumours may invade the serosal surface of the proximal stomach with or without CRM involvement of the lower oesophagus. Assessment for serosal invasion in this area is included in the oesophageal minimum dataset as there is strong evidence that it is a poor prognostic factor in gastric cancer [57]. However no studies have been performed to prove this is the case in oesophageal tumours [57].

#### Vascular and lymphatic vessel invasion (LVI)

Vascular invasion is known to be a strong prognostic factor and is included in the oesophageal minimum dataset. However, it is not included in the gastric minimum dataset [60], which further highlights the differences between the two proformas. Recent evidence has shown that LVI is a strong prognostic factor in both squamous cell carcinomas and adenocarcinomas of the oesophagus [61-63]. In a study focusing on GOJ adenocarcinomas it was found to be an independent prognostic factor [63]. Interestingly in this large study, it appeared to be more prognostic in type II and III GOJ tumours, compared with type I cancers. This adds to the argument that there are biological differences between these tumour types.

#### Lymph node metastases

The presence of lymph node metastases is often the most significant independent factor on multivariate analysis [42,43,64,65]. However, there is no consensus regarding minimum numbers of lymph nodes to be included in a curative resection for accurate pathological staging or on the sampling technique used. The minimum dataset for oesophageal carcinoma does not comment on how to sample lymph nodes [25]. The minimum dataset for gastric cancers states that the lymph node should be cut through its greatest diameter and one half taken for microscopy [60]. However, this sampling technique has the potential of missing metastatic deposits and it is best practice to examine the whole node microscopically unless it is clearly replaced by tumour [26].

The current (2002) UICC guidelines recommend a minimum examination of 6 lymph nodes to classify a patient N0 [66]. However this falls short of the 15 recommended by the consensus conference of the International Society for Diseases of the Esophagus (ISDE) [67]. As mentioned previously, the current oesophageal staging criteria simply divide patients into lymph node metastases present (pN1) and lymph node metastases absent (pN0). This system is crude and does not take into account the total number of resected/examined nodes. There is strong evidence that a lymph node ratio (number of nodes involved/number nodes examined) may be a better system. The prognostic significance of metastatic lymph node ratio has been described in oesophageal adenocarcinoma in Western patients (ratios of 0.2 and 0.3) [42,53,68], squamous cell carcinoma in Western patients (ratio of 0.2) [64] and squamous cell carcinoma in Japanese patients (ratio of 0.1) [69]. The differences in ratios for each of these studies may reflect the differences in nodal yields obtained from two-field oesophago-gastrectomy for adenocarcinoma and three-field oesophago-gastrectomy for squamous cell carcinoma. Noticeably in all these five studies the lymph node ratio was of greater prognostic significance than the N stage. As there is no consensus for the exact lymph node ratio, it remains important for the pathologist to accurately report the total number of involved nodes and the total number examined. Also at present only one level of each lymph node is examined microscopically. Further studies researching the benefit of further levels are required [70]; this may potentially yield additional prognostic information, especially in patients who are initially designated pN0 with a low yield of lymph nodes.

In addition to a pure number based system, nodal involvement in relation to the lymph node capsule (intra or extracapsular) has recently been shown to be strongly prognostic on multivariate survival analysis in oesophagogastric adenocarcinoma [71,72]. The 5-year survival for patients with intracapsular nodal involvement was 40.9% compared with only 18% with extracapsular involvement [72].

#### Immunohistochemically detected lymph node micrometastases

Immunohistochemical techniques can identify micrometastases which are missed by standard haematoxylin and eosin staining. Cytokeratin, a component of the cytoskeleton of epithelial cells, is not found in normal nodes enabling monoclonal antibodies to certain cytokeratin markers (such as AE1/AE3) to be used to detect micrometastases. These techniques may detect single tumour cells or cell clusters in lymph nodes that have been staged as tumour free on routine examination. The prognostic outcome of the detection of micrometastases detected by immunohistochemistry is controversial as some studies have found an association with increased risk of tumour recurrence and decreased survival [73-75], but others have not [76,77]. The viability of these tumour cells and their potential to form true metastases has been questioned. As such, these techniques remain research tools and are not currently used in daily clinical practice.

### Sentinel node concept in oesophageal and GOJ cancer

The importance and clinical utility of the sentinel node concept has been extensively evaluated in malignant melanoma and breast cancer. It is being evaluated in gastrointestinal cancers [78], including oesophageal cancer. The sentinel node concept relies on two assumptions. Firstly, lymphatic metastases from a solid tumour follow a predictable course and that there is always one node (the 'sentinel node') or group of nodes that is affected first. Secondly, metastases to other lymph nodes or lymph nodes groups cannot occur without involvement of the sentinel node. These assumptions remain to be conclusively proven in the context of oesophagogastric cancer and other confounding factors may exist, such as the possibility of 'skip' metastases [79] and the alteration of lymphatic flow due to 'blocked' nodes.

Initial feasibility studies have been performed in oesophageal cancer [78,80]. The principal benefit of the sentinel lymph node concept if validated in oesophageal cancer surgery may be to permit tailoring the extent of the lymphadenectomy. The morbidity from extensive lymphadenectomy can be high; therefore, if the sentinel node is not involved then patients could be spared more extensive surgery. However, the complex and extensive lymphatic drainage of the oesophagus may make this approach problematic and its ultimate role is likely to be limited. In addition, the technique relies on accurate pathological examination of the sentinel node intra-operatively which has its own drawbacks. The exact technique has yet to be defined, but frozen sectioning, touch imprint cytology and rapid immunohistochemistry are being evaluated. Until more extensive high quality prospective studies are performed in oesophageal cancer, the usefulness of the sentinel node concept in this area remains uncertain.

### Endoscopic mucosal resection (EMR)

Endoscopic mucosal resection (EMR) techniques are being increasingly used for treatment and staging of superficial early cancers of the oesophagus, especially in Japan although Western centres are gaining experience [81,82]. Although the precise indications for EMR have not been established, in the oesophagus EMR may be curative for small superficial squamous cell carcinomas or adenocarcinomas which are limited to the mucosa or lamina propria [83]. HGD of the oesophagus can also be treated with EMR techniques. The treatment of circumferential lesions is possible, but there is a high risk of subsequent stricture formation. The resected specimen must be carefully examined in its entirety for accurate pathological staging [84] and to allow future audit and preparation of appropriate guidelines on the safe use of this technique. The main problem with EMR is the lack of pathological lymph node staging. The risk of lymph node metastases increases with tumour penetration through the mucosa and submucosa [84]. Based on the Japanese classification of early neoplasia of the oesophagus a more comprehensive staging system than TNM is suggested when reporting such specimens. The recommended staging splits mucosal involvement into three categories (m1: equivalent to HGD with questionable invasion beyond the basement membrane; m2: invasion of the lamina propria and m3: invasion into but not through the muscularis mucosa) and submucosal involvement into 3 categories (sm1: invasion into the upper third of submucosa; sm2: invasion into the middle third of submucosa and sm3: invasion into the lower third of submucosa). Lymphovascular invasion should be reported if present. As some series report a high rate of incomplete resections with this technique, some authors advocate its use as a diagnostic and staging tool rather than a therapeutic technique [82].

### Multimodal therapy and implications for pathological specimen reporting

Multimodal therapy, in particular neo-adjuvant treatment prior to surgical resection, is increasingly used in oesophageal cancer. The aims of neo-adjuvant chemotherapy, with or without radiotherapy, are to downstage or 'sterilise' the primary to improve the likelihood of complete tumour (R0) resection, reduce tumour recurrence, treat occult micro-metastases and ultimately to improve overall survival. Although there is some evidence from randomised controlled trials that neo-adjuvant therapy in addition to surgery can prolong survival [85-88], there remains considerable debate in the literature about the benefits of therapy and the definitive regime has yet to be defined. Other authors have found that multimodal therapy is associated with an increased post-operative mortality [88,89] and morbidity [90,91], especially sepsis related complications, respiratory failure and adult respiratory distress syndrome.

A pathological complete response (CR) occurs in less than 30% of patients who undergo surgery after preoperative chemotherapy or chemo-radiotherapy [92-94]. Although patients who achieve a CR appear to have a longer overall survival [95-97] these results are mainly from sub-group analysis and should be treated with some caution.

#### Classifying pathological response to multimodal therapy

In instances of a potential CR, knowledge of the precise location of the tumour before neo-adjuvant therapy is crucial to direct pathological sampling as the oesophagus may look normal macroscopically. However, the precise number of tumour blocks to take has yet to be clarified and currently is at the discretion of the histopathologist. Various architectural, nuclear and cytoplasmic changes in tumour and non-tumour tissue have been described following neo-adjuvant chemotherapy [98]. In addition, radiotherapy induced changes include fibrosis, telangiectasia of submucosal vessels, bizarre nuclear appearances in tumour and stromal cells and necrosis [99]. Mucin lakes and collections of keratin are considered to represent areas where tumour has been present prior to sterilisation by chemotherapy. Preliminary evidence suggests that prominent acellular mucin pools in patients who have received neo-adjuvant chemotherapy should not be considered evidence of residual disease [100]. Immunohistochemistry using cytokeratin antibodies may be required to identify residual tumour cells not readily seen on haematoxylin and eosin staining.

There are a variety of different grading systems for measuring residual tumour in oesophageal resection specimens after preoperative chemotherapy, with or without radiotherapy (Table 6) [101-103]. The Mandard *et al*., system [103] would seem to be the most applicable because it assesses residual tumour in relation to background fibrosis, and this has been shown to be important in oesophageal cancer. However, this system was not shown to yield prognostic information in a recent study [98], whereas the residual carcinoma status correlates significantly with prognosis in several retrospective studies [101,104]. It also uses a simple system of percentage of residual tumour which is likely to be more reproducible. However, none of these systems have been universally accepted (Table 6).

**Table 6:** Classification systems to grade tumour response to neo-adjuvant chemo-radiotherapy

**Reference, Name of classification system**		**Details/definition**
Mandard et al [103]; Tumour regression grade (TRG)	TRG1	Complete pathological regression: absence of residual cancer and fibrosis extending through the layers of the oesophageal wall
	TRG2	Presence of rare residual cancer cells scattered through the fibrosis
	TRG3	Increase in the number of residual cancer cells, but fibrosis still predominant
	TRG4	Showing residual cancer outgrowing fibrosis
	TRG5	Absence of regressive changes
Chirieac et al [101]; Residual carcinoma status	0	No residual cancer
	1	1% to 10% residual cancer
	2	11%–50% residual cancer
	3	>50% residual cancer
General rules for oesophageal cancer proposed by the Japanese Society for Esophageal Disease [120] *	Complete response	Disappearance of the primary tumour in the postoperative specimen
	Partial response	Microscopic evidence of residual tumour in the postoperative specimen
	Stable disease	Less than 50% decrease or less than a 25% increase in tumour volume
	Progressive disease	No significant change in tumour mass or more than a 25% increase in tumour volume

*In addition to pathological assessment of the resection specimen, the designation to stable or progressive disease is by re-evaluation of the primary tumour by computed tomography and endoscopy 2 weeks after completion of CRT.

With the increased interest in multi-modal therapy, however, the classification of pathological response is of increasing importance to the histopathologist and surgeon. Proposals to revise the oesophageal cancer staging system to accommodate the pathological response of the tumour following preoperative chemo-radiotherapy have recently been made [104]. The final pathological staging in the cases treated with neo-adjuvant therapy should be prefixed 'y' (for example, ypT2, ypN0, ypM0) [34].

#### Predictive factors for response to neo-adjuvant chemo-radiotherapy

There is great interest in evaluating predictive factors for patient response to neo-adjuvant chemo-radiotherapy. An accurate predictive factor would allow the targeting of therapy to patients who are most likely to achieve a benefit, while those that are unlikely to respond can avoid potentially toxic therapy and receive earlier surgery.

A recent study has shown that patients with signet-ring cell or mucinous histology on pre-treatment biopsies have an improved response and better overall survival when treated with neo-adjuvant chemo-radiotherapy [105]. This study compared 193 patients who were treated with chemo-radiotherapy (5-Flurouracil, cisplatin and taxane with 45 Gy radiotherapy in 25 fractions) followed by surgery with 219 patients who had surgery alone. In the patients who had surgery alone, the overall survival rate was significantly worse if signet-ring or mucinous histology was present. However, in the patients who were treated with chemo-radiotherapy signet-ring or mucinous histology predicted a better overall survival.

More sophisticated molecular techniques have been evaluated on pre-therapeutic biopsies in an attempt to find a good predictive marker. Although, none of these are currently being used in routine clinical practice, the role of the histopathologist in this regard is likely to increase in the future.

### Multidisciplinary meetings

The management of patients with oesophagogastric cancer should be discussed at all key points in the patient journey at specialist multidisciplinary team (MDT) meetings [106]. There is some evidence that MDT discussion is associated with improved patient outcomes in oesophageal cancer [107]. Stephens et al reported a lower operative mortality and improved 5-year survival in R0 resected patients who were discussed at an MDT compared with patients who underwent R0 resection by independent surgeons [107]. The management of patients with oesophagogastric cancer is complex and involves input from several clinical specialities. A forum in which to review the histological slides may lead to alteration in the final pathological diagnosis [108], either due to specialist pathological review or by additional information provided by the clinician. Regular communication between all specialities at these meetings provides an opportunity to improve and maintain the quality of pathological reporting. Recent evidence has shown that MDT discussion improves the accuracy of clinical TNM stage allocation and ensures that correct management decisions are applied to patients with gastro-oesophageal cancer [109]. The MDT meeting may also allow the preoperative staging imaging to be compared with the histopathological report or images and this feedback facilitates training, audit and teaching.

## Conclusion

Accurate assessment of endoscopic biopsy material is crucial in the assessment of patients with Barrett's oesophagus. As appropriate care of patients with HGD in the setting of Barrett's oesophagus relies heavily on the accuracy of reporting the degree of dysplasia, standardised methods and guidelines should be followed.

The classification and staging systems for GOJ tumours need to be improved and future research into this area is greatly warranted. A better understanding of the clinical relevance of each classification system for GOJ tumours needs to be achieved before a final recommendation is made. Although the Siewert classification has been shown to have some clinical relevance, other authors have found that it unnecessarily complicates the assessment of these tumours and is fraught with difficulties because of the overlapping nature of tumours in this region. Some authors would argue that instead of three staging systems, only two are required (with a staging system optimised against current criticisms for oesophageal and GOJ tumours and a separate staging system for gastric tumours). Further clinical studies to address these issues are urgently required.

There is already sufficient evidence to confirm that CRM involvement is a marker of poor prognosis in oesophageal cancer. As such, CRM status must continue to be routinely reported. However, our understanding of its full significance is limited compared to the field of rectal cancer where CRM has been extensively studied. For example, studies have been carried out directly comparing histopathological sections with preoperative cross-sectional imaging. In rectal cancer, this information has been used to predict potential CRM involvement prior to surgery and thus the need for neo-adjuvant therapy. In future oesophageal studies, especially prospective trials involving neo-adjuvant therapy or the comparison of different surgical techniques, it is imperative that involvement of the CRM is analysed.

There are exciting new research opportunities in the identification of lymph node micrometastases and sentinel node involvement; however they have yet to be proven clinically useful. There is emerging evidence that the histopathological evaluation of the tumour response to neo-adjuvant therapy is prognostically relevant. However, further research studies are required to confirm its role in patient management. Although several classification systems have been devised they have yet to be agreed for routine clinical use. A standardised classification system for the assessment of residual tumour burden after neo-adjuvant therapy will need to be agreed and ideally adopted internationally.

## Conflict of interests

The author(s) declare that they have no competing interests.

## Authors' contributions

EG conceived of the original manuscript idea, performed the literature search and wrote the first draft. SP, NM and IW revised subsequent versions of the manuscript for intellectual content. All authors read and approved the final manuscript.
